# Switching from Kivexa + efavirenz to Atripla reduces total cholesterol in hypercholesterolemic subjects: final results of a 24-week, randomized study

**DOI:** 10.1186/1758-2652-13-S4-P80

**Published:** 2010-11-08

**Authors:** G Moyle, C Orkin, M Fisher, J Anderson, J Dhar, MH Wang, J Ewan

**Affiliations:** 1 Chelsea & Westminister Hospital, HIV/GU Medicine, London, UK; 2 St. Bartholomew's Hospital, HIV Department, London, UK; 3 Royal Sussex County Hospital, The Lawson Unit, HIV Department, Brighton, UK; 4 Homerton hospital, Sexual Health Clinic, London, UK; 5 Leicester Royal Infirmary, GU Medicine, Leicester, UK; 6 Gilead Sciences, Biostatistics, Foster City, USA; 7 Gilead Sciences Limited, Flowers Building, Granta Park, Cambridge, UK

## Background

Dyslipidemia in persons with HIV contributes significantly to cardiovascular (CV) risk. Abacavir (ABC) has been shown to increase lipid levels and some cohort studies have suggested an association between ABC use and myocardial infarction (MI). Comparative data suggested Truvada (TDF/FTC) has a lesser effect on lipid parameters than Kivexa [KVX]. We investigated the change in fasting lipid parameters in hypercholesterolemic subjects switching from KVX + Efavirenz [EFV] to Atripla [ATR].

## Methods

A 24-week, UK, open-label study in subjects stable on once daily (QD) KVX+EFV, HIV RNA <50 copies/mL for ≥6 months and fasting total cholesterol [TC] ≥5.2 mmol/L at screening, randomized to continue KVX+EFV or switch to QD ATR. The primary endpoint was change from baseline to Week 12 in fasting TC. Changes in fasting lipid parameters and 10 year risk score for coronary heart disease (CHD) were also assessed. At Week 12 subjects continuing on KVX+EFV were switched to ATR (delayed switch to ATR) and all subjects received ATR until Week 24.

## Results

**Figure 1:**
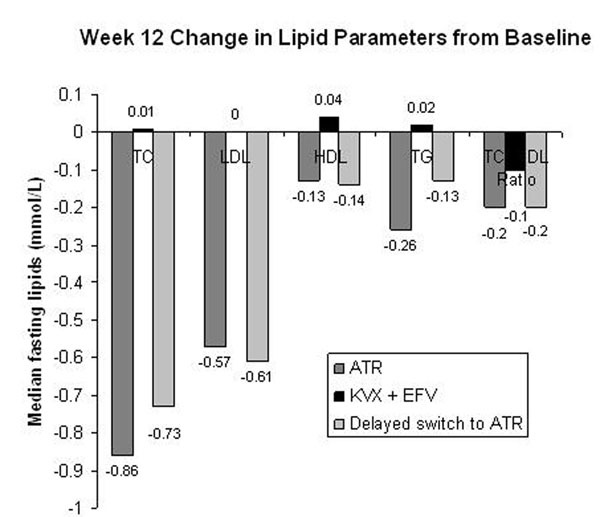


157 randomized subjects received at least 1 dose of study medication; 78 continued KVX+EFV, 79 switched to ATR at baseline; 69 switched to ATR at Week 12. Subjects were well matched for baseline characteristics. Figure 1.

At 12 weeks there was a significant reduction in TC, LDL, HDL and TG in the ATR arm (p<0.001) and the ATR vs KVX+EFV between group difference (p<0.001), which was confirmed for TC, LDL and HDL in the delayed switch to ATR arm after 12 weeks of switch. The mean (SD) change in 10-year risk for CHD was -0.6 (3.85) ATR vs -0.1 (2.69) KVX+EFV at Week 12 and -0.1 (3.68) in the delayed switch to ATR arm after 12 weeks of switch. There were no protocol defined virologic failures and no study drug related SAEs.

## Conclusion

Switching from KVX+EFV to ATR led to a significant, rapid decline in lipid parameters and this may have had a positive impact on calculated CHD risk while maintaining virologic suppression. The full study results showed that the initial 12 week results were replicated in the delayed switch to ATR arm. These results confirm that ATR is a preferred treatment option to a KVX based regimen in hypercholesterolemic patients.

